# Lichen elemental composition distinguishes anthropogenic emissions from dust storm inputs and differs among species: Evidence from Xilinhot, Inner Mongolia, China

**DOI:** 10.1038/srep34694

**Published:** 2016-10-04

**Authors:** Hua-Jie Liu, Shi-Bo Fang, Si-Wa Liu, Liang-Cheng Zhao, Xiu-Ping Guo, Yun-Jun Jiang, Jian-Sen Hu, Xiao-Di Liu, Yu Xia, Yi-Dan Wang, Qing-Feng Wu

**Affiliations:** 1 College of Life Sciences, Hebei University, Baoding, Hebei 071002, China; 2 State Key Laboratory of Severe Weather, Chinese Academy of Meteorological Sciences, Beijing, 100081, China; 3 Collaborative Innovation Center on Forecast and Evaluation of Meteorological Disasters, Nanjing University of Information Science & Technology, Nanjing, 210044, China; 4 Hebei Geological Laboratory, Baoding, Hebei 071051, China; 5 College of Chemistry and Environmental Sciences, Hebei University, Baoding, Hebei 071002, China

## Abstract

To test the applicability of lichens in the biomonitoring of atmospheric elemental deposition in a typical steppe zone of Inner Mongolia, China, six foliose lichens (*Physcia aipolia*, PA; *P. tribacia*, PT; *Xanthoria elegans*, XE; *X. mandschurica*, XM; *Xanthoparmelia camtschadalis*, XPC; and *Xp. tinctina*, XPT) were sampled from the Xilin River Basin, Xilinhot, Inner Mongolia, China. Twenty-five elements (Al, Ba, Cd, Ce, Cr, Cs, Cu, Fe, K, La, Mn, Mo, Na, Ni, P, Pb, Sb, Sc, Sm, Tb, Th, Ti, Tl, V and Zn) in the lichens were analysed using inductively coupled plasma mass spectrometry (ICP-MS). The results show that Cd, Pb and Zn were mainly atmospheric in origin, whereas the other elements were predominantly of crustal origin. Compared with other studies, our data were higher in crustal element concentrations and lower in atmospheric element concentrations, matching with the frequent, severe dust storms and road traffic in the area. The elemental concentrations in lichens are both species- and element-specific, highlighting the importance of species selection for biomonitoring air pollution using lichens. We recommend PT, XE, XM and XPT for monitoring atmospheric deposition of crustal elements; XPC and XPT for Cd and Pb; PA for Cd and Zn; and PT for Cd.

Atmospheric metal deposition has become a major environmental problem in China due to its adverse effects on human and ecosystem health^1^,^2^. This is particularly true in Inner Mongolia, where frequent dust storms (due to steppe degradation and desertification) and air pollution (associated with the increasing urbanization and industrialization) have occurred in recent decades. However, there are insufficient data to fully evaluate this problem in the region. Although air pollution monitoring systems based on traditional (instrumental) methods have been established in Inner Mongolia, these methods have a low sampling density in space and only monitor a limited number of pollutants (mainly CO, SO_X_, NO_X_ and dust). Studies on the atmospheric deposition of elements in Inner Mongolia have mainly focused on nitrogen^3^,^4^.

Lichens can be a complement, or to a lesser extent, an alternative to the traditional methods of studying atmospheric deposition from anthropogenic or natural sources and have been widely used outside of China in the past few decades^5^,^6^. These organisms are highly dependent on atmospheric deposition for mineral nutrients and have the extraordinary capability of accumulating metals far above their need. These features of lichens, combined with their widespread distribution and long lifespan, rank them among the best bioindicators and biomonitors of air pollution^5^,^6^. Although lichens are widespread in the diverse ecosystems of Inner Mongolia, they have not been applied to air quality assessment in the region.

Two questions are prerequisite to the assessment of air quality in Inner Mongolia using lichens. (1) Can lichens distinguish anthropogenic contribution from dust storm inputs? This question has rarely been addressed because most relevant studies have been performed in industrial/urban or remote sites without dust storms^7^,^8^,^9^,^10^,^11^,^12^,^13^. (2) Which lichen should be selected as a biomonitor? Species selection is necessary in such studies because the metal concentrations and enrichment capability are different among lichens^5^,^14^,^15^,^16^.

We sampled six foliose lichens (*Physcia aipolia*, PA; *P. tribacia*, PT; *Xanthoria elegans*, XE; *X. mandschurica*, XM; *Xanthoparmelia camtschadalis*, XPC; and *Xp. tinctina*, XPT) from a remote region of the Xilin River Basin (XILRB), one of the most representative geographic areas of the Inner Mongolia steppe zone with frequent dust storms (Fig. 1). Twenty-five elements (Al, Ba, Cd, Ce, Cr, Cs, Cu, Fe, K, La, Mn, Mo, Na, Ni, P, Pb, Sb, Sc, Sm, Tb, Th, Ti, Tl, V and Zn) were measured. The purposes of the present study were to quantify the concentration and identify the origin of elements in lichens and to select suitable lichens for monitoring atmospheric deposition of mineral elements in Inner Mongolia.

## Results

**Figure 1:**
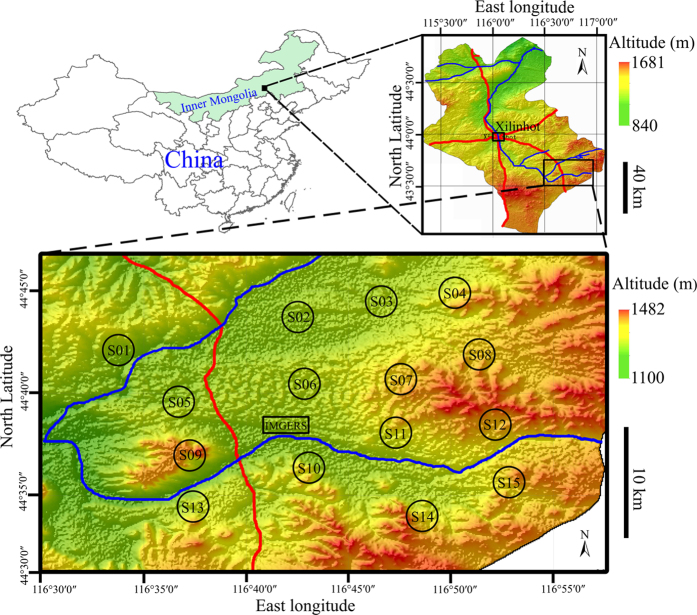
Location of the sampling sites. The black hollow circles denote the sampling sites. The red and blue lines represent main roads and rivers, respectively. The map was prepared using ArcGIS 10.2 software (ESRI, Inc., Redlands, CA, USA; http://www.esri.com/) with 3D visualization of the DEM data to illustrate the geomorphic features. The rivers and roads were drawn according to Li *et al.*^36^ by hand. IMGERS denote Inner Mongolia Grassland Ecosystem Research Station, Chinese Academy of Sciences. For details of the DEM data, see the Methods section.

Six lichens were sampled from 15 sampling sites (Fig. 1). There were no significant differences in the mean distance from the sampling sites to the national road between these lichens (XPC: 10.7 ± 5.3 km; PA: 10.6 ± 5.3; PT: 10.5 ± 6.4; XPT: 10.4 ± 6.4; XM: 10.4 ± 6.3; XE: 9.3 ± 6.3. All at p > 0.10; One-way analyses of variance).

### Element concentration and correlation

The concentrations of 25 elements in lichens and surface soil (SS) are given in Table 1. One-sample Kolmogorov-Smirnov tests show that the concentrations of each element are normally distributed across lichens and within each lichen species (all at p > 0.05). The concentrations were distributed in the following order across all lichens: K > Al > Fe > P > Na > Ti > Mn > Ba > Zn > Cr > V > Ce > Pb > Cu > Ni > La > Cs > Th > Sc > Sm > Mo > Sb > Cd > Tl > Tb. This order is roughly followed in each lichen (Table 1).

**Figure 2:**
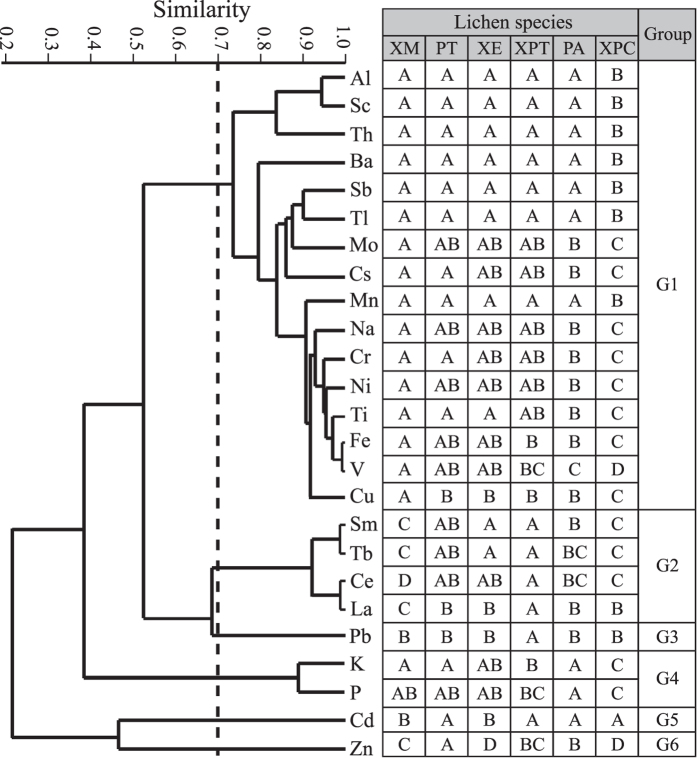
Dendrogram obtained by using UPGMA cluster analysis based on the correlation distance matrix (left panel) and the Table showing differences in the element concentrations between lichens (right panel). The broken line in the dendrogram shows a correlation similarity of 0.70. Different capitalized letters in each row of the table indicate a significant difference in element concentration between lichens (p ≤ 0.05). Cluster analysis was performed on the raw concentrations, and the resultant cophenetic correlation index was 0.839. Species differences were evaluated from the log10-transformed concentrations using one-way ANOVA followed by *post hoc* SNK tests. All tests were performed on 37 lichen samples from 15 sites in a remote region of Xilin River Basin, Xilinhot, Inner Mongolia, China. XM refers to *Xanthoria mandschurica* (n = 5). PT refers to *Physcia tribacia* (n = 5). XE refers to *Xanthoria elegans* (n = 6). XPT refers to *Xanthoparmelia tinctina* (n = 5). PA refers to *Physcia aipolia* (n = 6). XPC refers to *Xanthoparmelia camtschadalis* (n = 10).

Concentration correlations between elements across all lichens were tested using cluster analysis (Fig. 2) and Pearson correlation tests. Cluster analysis preserved most of the pairwise distances between the original unmodelled data points (cophenetic correlation coefficient = 0.839) and distinguished six groups at a correlation similarity of 0.70. Group G1 contains 16 metals (Al, Ba, Cr, Cs, Cu, Fe, Mn, Mo, Na, Ni, Sb, Sc, Th, Ti, Tl and V). G2 contains 4 lanthanoids (Ce, La, Sm and Tb). G3 contains Pb. G4 contains K and P, which are strongly correlated (r = 0.89, p = 0.000; Pearson correlation test). Cd and Zn are not strongly correlated with other elements and formed G5 and G6, respectively (Fig. 2).

### Enrichment factor (EF)

To assess the relative importance of crustal input in lichen elemental composition, element concentrations were normalized to Al in SS and in upper continental crust (UCC) to obtain EF_SS_ and EF_UCC_, respectively, according to Eq. 1. The results are given in Table 2. Both EF_SS_ and EF_UCC_ were >5 for three elements (Cd, P and Zn) across all lichens and in each lichen, as well as for Pb in XPT and XPC. Other metals were mostly <5 in mean EF_SS_ (Table 2). Differences in the elemental ratios between SS and UCC resulted in marked differences between EF_SS_ and EF_UCC_ in some cases. For example, K (in PA and XPC) and Sb (in all lichens) had an EF_UCC_ of >5 but an EF_SS_ of <5 (Table 2).

### Element concentration differences between lichens

One-way analyses of variance (ANOVA) based on the log10-transformed concentrations show that all elements were significantly different among lichens (all significant at p < 0.05). *Post hoc* Student-Newman-Keuls (SNK) tests show that the element concentrations are dependent on lichen species and elements (Table 1). For the sake of clarity and simplicity, the SNK results were combined in Fig. 2. Figure 2 shows that elements that showed strong positive correlations had similar lichen patterns; our results can therefore be generalized at the group scale.

The concentrations of the G1 metals (except Cu) were generally highest in PT, XE and XM, which were similar to XPT for 13 metals (Al, Ba, Cr, Cs, Mn, Mo, Na, Ni, Sb, Sc, Th, Ti and Tl) and to PA for 7 metals (Al, Ba, Mn, Sb, Sc, Th and Tl). PA was moderate in the concentrations of 8 metals (Cr, Cs, Fe, Mo, Na, Ni, Ti and V), among which Fe and V were also moderate in XPT. The Cu concentration was the highest in XM and was not significantly different between those of PA, PT, XE and XPT. The concentrations of all of the G1 metals were lowest in XPC (Fig. 2, Table 1).

The concentrations of the G2 metals were highest in XPT, which was close to PT and XE for Ce, Sm and Tb. XM was lowest for lanthanoids, among which Sm and Tb were also lowest in XPC. The Pb concentrations were not significantly different among lichens, except XPT. The concentrations of the G4 elements were highest in XM, PT, XE and PA and were lowest in XPC. XM and XE were significantly lower in the concentration of Cd than other lichens. The Zn concentration was highest in PT, followed by PA, XM and XPT and was lowest in XE and XPC (Fig. 2).

### Comparison with the Taihang Mountains

**Figure 3:**
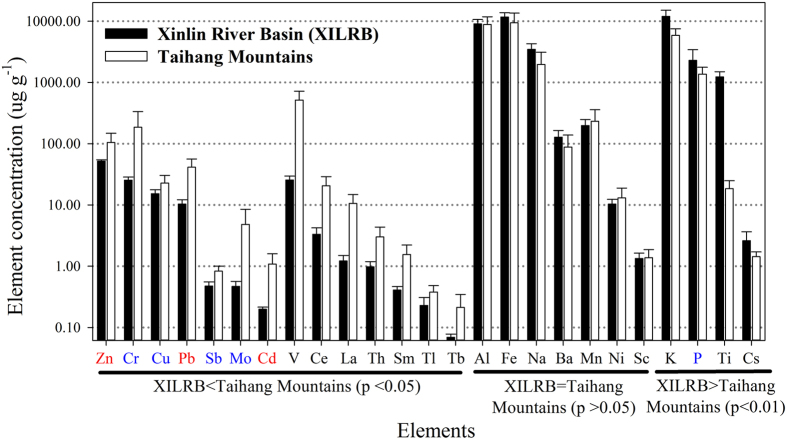
Element concentrations in XM from XILRB and Taihang Mountains. Elements in red and in blue are of atmospheric origin in both regions and only in the Taihang Mountains, respectively. Elements in black are crustal elements in both regions. For detailed information on elemental concentrations in XM (*Xanthoria mandschurica*) from XILRB, see Table 1. Data from the Taihang Mountains were adopted from Liu *et al.*^20^.

A comparison of the element concentrations in XM between XILRB and the Taihang Mountains is presented in Fig. 3. Compared with the Taihang Mountains, XILRB was significantly higher in concentrations for 4 elements (68–104% higher for Cs, K and P, and 65.6 times higher for Ti; all significant at p < 0.05, one-way ANOVA) and lower in concentrations for 14 metals (33–88% lower for Cd, Ce, Cr, Cu, La, Pb, Sb, Sm, Tb, Th, Tl and Zn; 90% lower for Mo and 95% lower for V; all significant at p < 0.05, one-way ANOVA). Seven metals (Al, Ba, Fe, Mn, Na, Ni and Sc) are not different in concentration between the two regions (all p > 0.05, One-way ANOVA).

## Discussion

### Elemental sources

Generally, our results show that Cd, Pb and Zn are most likely of atmospheric origin, most likely from road traffic, whereas the other elements are mainly of crustal origin and are associated with dust storms.

The EF of an element provides information regarding its origin. An EF of >5 often indicates a non-crustal or non-local source input, whereas an EF of <5 is evidence of crustal input^17^. However, due to the different elemental ratios between SS and UCC, some metals had an EF_UCC_ of >5 but an EF_SS_ of <5, such as for K (in PA and XPC) and Sb (in all lichens; Table 2). We suggest that local SS is more applicable as a background reference than UCC because the suspension of SS is most likely the main source of atmospheric particulates due to frequent and severe dust storms in the Inner Mongolia steppe zone.

Both Cd and Zn were high in EFs (EF_SS_ and EF_UCC_ > 10 across all lichens; EF_SS_ > 5 in each lichen; Table 2), suggesting that other sources besides local crustal input can have a remarkable influence on the atmospheric deposition of both metals. This is also true for Pb, which had an EF_SS_ of >5 in XPC and XPT. A large body of relevant literature has attributed the enrichment of these metals in lichens to anthropogenic pollutants released from industrial activities or traffic^5^,^9^,^11^,^13^,^17^,^18^,^19^,^20^. However, this region is a rural, pastoral area without intense industrial operations and is far from range fires and the application of chemical fertilizers and pesticides. The most possible source of these metals may be related to a national road crossing the study area (Fig. 1). Lichens near roads often accumulate high levels of traffic-related metals, particularly Cd, Pb and Zn^5^. The Pb concentration in lichens has decreased as a result of declined vehicular Pb emissions due to the use of unleaded gasoline^5^. Accordingly, Pb was only slightly enriched in two lichens and the EF_SS_s (5.4–7.0) were much lower than those of Cd and Zn (Table 2). Resuspension of “old” Pb in soils may be an important source of Pb. Atmospheric pollutant transportation from the nearby city of Xilinhot (50 km away from our sites; Fig. 1) may also be a source of these metals.

Although both the EF_SS_ and EF_UCC_ of P were > 10 (Table 2), a strong positive correlation between P and K (Fig. 2) suggests that the enrichment of P is not necessarily evidence of a non-crustal input but may be the result of active absorption and/or bio-regulation because both elements are essential nutrients for plants. The other metals are mainly of crustal origin (EF_SS_s of <5), in strong agreement with many studies outside China^8^,^21^,^22^,^23^,^24^,^25^.

### Elemental concentration

Lichens in this study had higher concentrations of crustal elements but lower concentrations of atmospheric elements compared with relevant studies, particularly those performed in “polluted” regions. These results match well with the facts of frequent, heavy dust storms and low anthropogenic emissions in this remote steppe.

A comparison with values from different lichens in diverse ecosystems shows that our data are higher than or at the upper range of the literature values for 16 crustal metals (Al, Ba, Ce, Cs, Fe, K, La, Mn, Na, P, Sc, Sm, Tb, Th, Ti and V)^7^,^8^,^9^,^12^,^18^,^21^,^22^,^23^,^24^,^25^,^26^,^27^,^28^,^29^,^30^,^31^,^32^. To minimize species effects, XM was compared between XILRB and a region of the Taihang Mountains where the air quality has long been influenced by local anthropogenic emissions and heavy air pollution in the adjacent North China Plain^20^. The results also show higher or similar concentrations in XILRB for 11 crustal elements (Al, Ba, Cs, Fe, K, Mn, Na, Ni, P, Sc and Ti; Fig. 3), clearly indicating a high level of crustal input in this region. By contrast, the literature records for 7 metals (Cd, Cu, Mo, Ni, Pb, Sb and Zn) are often 2 times to two orders of magnitude higher than our data, particularly at “polluted” sites where these metals were related to anthropogenic emissions^10^,^11^,^13^,^17^,^18^,^19^,^31^,^33^,^34^,^35^. Similarly, XM from the Taihang Mountains accumulated 1.5–10.3 times as much of these metals as XM from XILRB (Fig. 3), indicating a much lower level of anthropogenic emissions in XILRB. In addition, XM had higher K and P concentrations in XILRB than in the Taihang Mountains, suggesting a better physiology of lichens because the K-P association is often related to the nutrient conditions in plants.

### Species difference

It has been suggested that the response of lichen elemental composition to air pollution is different among lichen species and is dependent on the morphology and ecology of lichens^5^,^15^,^17^. This pattern is shown in our study, as indicated by the species- and element-specific concentrations (Table 1, Fig. 2). Therefore, it is important to select lichens according to the elements of interest.

The higher concentrations of crustal elements indicate a greater efficiency of entrapment and/or retention of particulates derived from crustal input. In this case, the four epilithic lichens (PT, XE, XM and XPT) are suitable for the assessment of the crustal input, because they were at the upper range of concentrations for most crustal elements. The concentration of elements in lichens could be related to the substrate type and intimacy of connection between lichen thalli and the substrate^6^,^21^. Generally, the epiphytic lichen PA was lower in concentration of crustal elements than the four epilithic lichens (PT, XE, XM and XPT; Table 1, Fig. 2), apparently due to the lower availability of aeolian particulates or soil/rock materials on bark relative to on rocks. Although it has been suggested that epigeal lichens are higher in crustal element concentrations than epiphytic lichens^7^,^30^, we found that the epigeal lichen XPC had the lowest concentrations of all of the crustal elements (Table 1, Fig. 2). The most possible explanation for these low concentrations is that the vagrant thalli of XPC are mostly free from the substrate and therefore have a lower degree of soil contact; by contrast, other lichens are closely adnate to the substrate.

Because Cd, Pb and Zn were mainly of atmospheric in origin, the higher values of EF_SS_ can be a measure of greater enrichment capability of lichens for these metals. XPT and XPC are the most suitable lichens for monitoring the atmospheric deposition of Pb. Pb was only enriched in these two lichens (EF_SS_ > 5; Table 2), and was constant in concentration in both lichens (CV < 20%; Table 1). Four lichens (PA, PT, XPC and XPT) are applicable for the monitoring of Cd. They had a greater enrichment capability of atmospheric Cd than other lichens, as indicated by the high concentration and EF_SS_ of Cd. PA is applicable to monitoring the atmospheric deposition of Zn. Although at the upper range of Zn concentration across all lichens, PT had a poor replicability in Zn concentration (CV = 50.3%) compared with PA (17.6%; Table 1). This could be explained by a higher degree of soil contamination in the thalli of the former lichen because the soils were spatially heterogeneous in Zn concentration (CV = 76.2% in SS; Table 1). Differences in accumulation of Cd, Pb and Zn between lichens are apparently not attributable to the location of sampling sites, because the mean distance from the sampling sites to the national road for each lichen ranged between 9.3 and 10.7 km and were not significantly different between lichens (All at p > 0.10; One-way ANOVA).

Both XE and XM are less applicable to monitoring atmospheric deposition of Cd, Pb and Zn. They had a lower enrichment capability of atmospheric metals than other lichens, as indicated by the lower EF_SS_s and concentrations (Tables 1 and 2). Although the applicability of XM in the biomonitoring of natural and anthropogenic emissions has been reported in the Taihang Mountains^20^, care should be taken in this region because the relative importance of anthropogenic contributions may be obscured by the high crustal input due to frequent dust storm events.

Our results show that anthropogenic emissions can be distinguished from the substantial dust storm input by analysis of the lichen elemental composition and therefore validate the applicability of lichens in monitoring atmospheric elemental deposition in ecosystems with prevailing dust storms. Different lichens should be selected according to the element of interest, for which a clear understanding of the species- and element-specific response of lichen elemental composition to atmospheric deposition is a prerequisite.

## Methods

### Study area

The study was conducted in the Inner Mongolia Grassland Ecosystem Research Station (IMGERS), the Chinese Academy of Sciences. The study area (43°32′–43°47′N, 116°33′–116°54′E) is located in the XILRB, Xilinhot, Inner Mongolia, China (Fig. 1). The climate is continental, temperate and semi-arid, with rare annual precipitation (350 mm, mostly occurs as rainfall during May-September) and frequent drought and windy episodes during the whole winter-spring season. The area has undergone steppe degradation and frequent dust storms due to the continuous drought, overgrazing and heavy wind erosion of soil in the past decades^36^. The dominant vegetation is typical steppe of the Inner Mongolia Plateau, with chestnut soil as the dominant soil type, interspersed with sand dune complexes with sandy soils dominant. The landforms are hills, lava tablelands and sandy lands, with an average elevation of 1,350 m^36^. There are two small villages near IMGERS, with a population of <100 inhabitants, and a national road crosses the area. The nearest city of Xilinhot is approximately 50 km northwest and has a population of 150,000 inhabitants (Fig. 1). Livestock grazing is the predominant type of agricultural operation. There are few farms, and the use of chemical fertilizers and pesticides is absent. No industrial activity was found in the area.

### Sampling sites

Fifteen sampling sites were selected at an interval of 4–8 km around IMGERS and covers approximately 260 km^2^ (Fig. 1). To minimize the potential effects of local anthropogenic emissions on the lichen elemental composition, each site was >1 km from major roads and settlements and >0.5 km from farms. Vegetation was mainly semi-fixed sand dune steppe (dwarf shrub and grass complex) in three sites (S02, S05 and S06), predominantly typical steppes in four sites (S04, S08, S09 and S14), and both vegetation types in eight sites (S01, S03, S07, S10, S11, S12, S13 and S15). Six foliose lichens were sampled in these sites, including four epilithic lichens (PT, XE, XM and XPT), one epigeal lichen (XPC) and one epiphytic lichen (PA, on barks of *Ulmus pumila*).

### Sampling strategies

Lichens and soils were sampled over the course of 10 days in September 2013. At each site, in an area of 1 km^2^, >50 mature individuals of each lichen and >10 subsamples of SS (0–10 mm soil) were randomly collected from diverse microhabitats, and the composite samples were mixed to represent the average status of the elemental composition in the site. The purpose of these protocols was to minimize the effects of the microhabitat and individual differences. A total of 37 samples of lichens were sampled. PA was sampled in 6 sites (S02, S03, S05, S07, S11 and S12), PT in 5 sites (S01, S08, S13, S14 and S15), XE in 6 sites (S01, S04, S09, S13, S14 and S15), XM in 5 sites (S01, S04, S13, S14 and S15), XPC in 10 sites (S01, S03, S04, S05, S06, S08, S10, S11, S12 and S13), and XPT in 5 sites (S01, S04, S08, S13 and S14; Fig. 1). Samples were transported to the laboratory in paper bags.

### Sample preparation and chemical analysis

Lichen thalli were carefully cleaned, and the outermost part (1–1.5 cm) was detached for analysis. We think that these parts could allow for the comparison of recently accumulated elements by greatly minimizing the effects of age^14^. Lichen samples were not washed to avoid element leaching and were oven-dried at 70 °C for 72 h to a constant weight. SS samples were thoroughly cleaned and dried using the above method. All samples were ground and homogenized in a grinding mill equipped with Tungsten Carbide jars (Retsch MM400; Retsch GmbH, Haan, Germany).

Each sample (200–300 mg) was mineralized by microwaves in high pressure Teflon vessels in a mixture of HNO_3_ and H_2_O_2_ for lichens, as well as in a mixture of HNO_3_, HF, HCl, and HClO_4_ for SS samples. Twenty-five elements (Al, Ba, Cd, Ce, Cr, Cs, Cu, Fe, K, La, Mn, Mo, Na, Ni, P, Pb, Sb, Sc, Sm, Tb, Th, Ti, Tl, V and Zn) were analysed at Hebei Geological Laboratory by inductively coupled plasma mass spectrometry (ICP-MS; Agilent 7700X; Agilent Technologies, Tokyo, Japan). The analytical quality of the results was evaluated against the following reference materials: the national reference materials of GBW07451, GBW07452 and GBW07457 for SS, as well as GBW10014 (cabbage), GBW10015 (spinach), GBW10052 (green tea; all materials mentioned above were issued by the Institute of Geophysical and Geochemical Exploration, Chinese Academy of Geological Sciences) and IAEA-336 (lichen, issued by the International Atomic Energy Agency) for lichens. The measured element concentrations were within the confidence intervals of the certified/suggested values. Analytical precision was generally <8% as judged from frequent, duplicate analyses.

### Calculation of EF

EF has been widely used in distinguishing element sources in lichens^8^,^17^,^20^,^25^. It is described in Eq. 1.


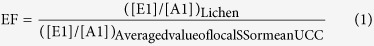


where “Al” is the chosen reference element in accordance with most relevant studies^8^,^20^,^25^. “El” is the element under consideration. The square brackets denote concentrations. The subscripts denote the sample type. The natural abundance of elements for the UCC standard was adopted from data summarized by Rudnick and Gao^37^.

### Statistical analyses

The normality of the data distribution was determined by the one-sample Kolmogorov-Smirnov test for the concentration of each element. CV was calculated as the standard deviation divided by the mean for each element and expressed as a percentage. Concentration correlations of lichen elements were tested by a Pearson correlation analysis and a cluster analysis. Cluster analysis was performed using the correlation distance to measure dissimilarities, and the UPGMA criterion was used to construct the hierarchical tree. All of the abovementioned analyses were performed using the Past 3.12 software (Ø. Hammer, Feb. 2016).

Concentration differences between lichens for each element were tested using one-way ANOVA based on the log10-transformed concentrations to satisfy the criteria of homogeneity and normality. *Post hoc* SNK tests were used for multicomparison. These analyses were performed using SPSS 13.0 (SPSS Inc., Chicago, IL, USA).

The maps in Fig. 1 were generated from the digital elevation model (DEM) data using ArcGIS 10.2 software (ESRI, Inc., Redlands, CA, USA; http://www.esri.com) for 3D visualization. The DEM data were obtained from the Geospatial Data Cloud (http://www.gscloud.cn/) and derived from the ASTER Global Digital Elevation Model (ASTER GDEM) with a horizontal accuracy of 30 m. The rivers and roads were drawn after Li *et al.*^36^ by hand.

## Additional Information

**How to cite this article**: Liu, H.-J. *et al.* Lichen elemental composition distinguishes anthropogenic emissions from dust storm inputs and differs among species: Evidence from Xilinhot, Inner Mongolia, China. *Sci. Rep.* **6**, 34694; doi: 10.1038/srep34694 (2016).

## Figures and Tables

**Table 1:** Concentrations of 25 elements in lichens from Xilin River Basin, Xilinhot, Inner Mongolia, China.

Element	Statistics	Concentration (μg·g^−1^)							
		Lichen species							SS
		All lichens	XM	PT	XE	XPT	PA	XPC	
Al	Mean	7293	9058A	11264A	10340A	8621A	6998A	3267B	42651
	CV(%)	53.12	18.11	33.18	32.17	34.91	29.97	38.96	18.95
Ba	Mean	79.15	127.8A	93.20A	95.18A	79.05A	116.5A	34.51B	625.2
	CV(%)	50.62	28.77	22.37	17.52	9.55	33.00	43.29	6.79
Cd	Mean	0.27	0.20B	0.33A	0.20B	0.35A	0.28A	0.27A	0.08
	CV(%)	25.41	10.00	12.12	15.00	25.71	21.43	11.11	45.92
Ce	Mean	9.64	3.33D	11.34AB	12.42AB	16.80A	8.58BC	6.67C	23.74
	CV(%)	49.65	27.03	24.78	33.33	31.55	15.50	27.14	56.44
Cr	Mean	14.15	25.39A	19.92A	17.77AB	16.41AB	12.71B	6.92C	337.5
	CV(%)	47.14	12.49	13.86	18.18	18.46	37.06	34.54	40.92
Cs	Mean	1.49	2.62A	2.30A	1.89AB	1.76AB	1.29B	0.66C	3.77
	CV(%)	53.71	39.31	16.96	22.22	30.11	28.68	37.88	65.29
Cu	Mean	8.84	15.25A	10.55B	10.20B	9.39B	9.52B	5.28C	19.31
	CV(%)	36.74	16.13	15.55	13.73	17.57	20.27	19.70	38.36
Fe	Mean	5926	11736A	8540AB	8226AB	6963B	5567B	1981C	11050
	CV(%)	58.50	17.24	23.83	22.88	23.61	31.39	30.47	66.15
K	Mean	8361	11981A	11596A	9374AB	6979B	11870A	4423C	23580
	CV(%)	44.77	25.99	20.97	29.97	9.20	24.06	23.69	3.62
La	Mean	5.08	1.22C	5.63B	6.23B	9.43A	4.29B	3.83B	12.90
	CV(%)	52.58	22.95	28.06	39.00	29.06	15.15	29.24	54.82
Mn	Mean	122.1	197.9A	176.2A	140.8A	133.3A	135.7A	59.82B	266.8
	CV(%)	47.75	25.45	32.16	17.99	29.47	34.08	23.24	90.61
Mo	Mean	0.31	0.47A	0.40AB	0.36AB	0.39AB	0.30B	0.18C	40.96
	CV(%)	39.67	21.28	35.00	25.00	12.82	23.33	22.22	20.84
Na	Mean	1650	3470A	2253AB	2404AB	1916AB	1437B	562.6C	12675
	CV(%)	60.82	23.24	20.05	23.36	16.43	31.94	57.14	7.72
Ni	Mean	5.90	10.32A	7.88AB	7.34AB	6.93AB	5.65B	2.93C	292.7
	CV(%)	46.41	19.86	30.08	24.52	15.73	22.12	28.67	19.26
P	Mean	1669	2297AB	2223AB	1799AB	1386BC	2511A	914.2C	258.7
	CV(%)	50.23	49.24	20.46	52.10	39.00	22.93	20.63	88.36
Pb	Mean	9.64	10.32B	11.07B	9.53B	14.85A	8.20B	7.47B	15.09
	CV(%)	31.12	18.22	13.46	16.05	17.44	29.27	19.54	29.70
Sb	Mean	0.30	0.48A	0.41A	0.34A	0.34A	0.36A	0.14B	0.60
	CV(%)	46.32	16.67	19.51	17.65	14.71	36.11	28.57	119.73
Sc	Mean	0.92	1.34A	1.37A	1.25A	1.07A	0.95A	0.39B	3.11
	CV(%)	53.87	22.39	40.15	33.60	33.64	23.16	41.03	80.67
Sm	Mean	0.76	0.41C	0.92AB	1.11A	1.22A	0.67B	0.47C	2.17
	CV(%)	48.18	14.63	23.91	30.63	29.51	14.93	27.66	59.06
Tb	Mean	0.10	0.07C	0.12AB	0.15A	0.16A	0.09BC	0.06C	0.30
	CV(%)	45.55	14.29	16.67	33.33	25.00	22.22	33.33	57.80
Th	Mean	1.30	0.98A	1.92A	2.19A	1.73A	1.29A	0.51B	3.85
	CV(%)	66.09	21.43	43.23	42.01	53.18	17.83	45.10	56.00
Ti	Mean	619.5	1232A	966.1A	876.3A	751.6AB	530.8B	182.9C	1241
	CV(%)	65.38	21.37	36.88	28.09	29.31	34.77	36.46	64.10
Tl	Mean	0.15	0.23A	0.21A	0.19A	0.17A	0.17A	0.08B	0.75
	CV(%)	44.23	34.78	23.81	31.58	11.76	23.53	12.50	9.09
V	Mean	13.11	25.61A	18.95AB	17.46AB	15.62BC	12.41C	4.69D	25.19
	CV(%)	56.04	15.62	20.63	20.33	20.17	34.09	28.14	78.52
Zn	Mean	56.28	51.96C	102.1A	39.28D	57.09BC	72.74B	38.19D	26.07
	CV(%)	51.22	4.93	50.26	7.03	8.15	17.60	18.83	76.21

Different capitalized letters in each row indicate a significant difference in element concentration between lichens (p ≤ 0.05) based on one-way ANOVA performed on the log10-transformed concentrations followed by *post hoc* SNK tests. CV refers to the coefficient of variance (defined as the standard deviation divided by the mean). XM refers to *Xanthoria mandschurica* (n = 5). PT refers to *Physcia tribacia* (n = 5). XE refers to *Xanthoria elegans* (n = 6). XPT refers to *Xanthoparmelia tinctina* (n = 5). PA refers to *Physcia aipolia* (n = 6). XPC refers to *Xanthoparmelia camtschadalis* (n = 10). SS refers to surface soil (n = 15). n = 37 for all lichens.

**Table 2:** EFs of 24 elements in lichens from Xilin River Basin, Xilinhot, Inner Mongolia, China.

Element	EFs	Lichen species													
		All lichen		XM		PT		XE		XPT		PA		XPC	
		EF_SS_	EF_UCC_	EF_SS_	EF_UCC_	EF_SS_	EF_UCC_	EF_SS_	EF_UCC_	EF_SS_	EF_UCC_	EF_SS_	EF_UCC_	EF_SS_	EF_UCC_
Ba	Mean	0.78	1.49	1.00	1.90	0.60	1.13	0.66	1.26	0.71	1.34	1.13	2.15	0.73	1.39
	CV(%)	32.79		43.01		27.33		19.35		42.23		9.75		18.69	
Cd	Mean	27.14	46.39	11.79	20.15	16.73	28.60	11.10	18.98	22.96	39.24	22.22	37.98	47.53	81.24
	CV(%)	61.57		11.10		27.96		35.96		30.70		32.81		23.75	
Ce	Mean	2.76	1.99	0.69	0.50	1.85	1.33	2.20	1.58	3.65	2.63	2.29	1.65	3.81	2.74
	CV(%)	42.63		38.56		8.22		22.49		31.63		19.56		17.13	
Cr	Mean	0.27	1.87	0.36	2.52	0.24	1.65	0.23	1.61	0.28	1.94	0.23	1.59	0.29	2.03
	CV(%)	36.11		16.81		21.35		23.46		47.80		19.82		42.42	
Cs	Mean	2.39	3.52	3.45	5.08	2.42	3.55	2.13	3.13	2.43	3.57	2.10	3.09	2.38	3.50
	CV(%)	32.94		55.15		16.77		13.41		24.54		12.32		35.77	
Cu	Mean	3.08	4.06	3.74	4.92	2.17	2.86	2.36	3.12	2.72	3.59	3.09	4.07	3.80	5.01
	CV(%)	31.32		8.23		18.86		32.16		44.67		19.27		20.59	
Fe	Mean	3.11	1.68	5.11	2.76	3.03	1.63	3.26	1.76	3.56	1.92	3.07	1.66	2.40	1.29
	CV(%)	35.93		26.81		19.04		30.60		51.27		14.20		14.03	
K	Mean	2.38	4.62	2.37	4.59	2.05	3.97	1.93	3.75	1.62	3.15	3.21	6.21	2.65	5.15
	CV(%)	40.28		8.68		44.95		62.12		36.61		29.19		30.24	
La	Mean	2.74	2.18	0.46	0.37	1.68	1.34	2.00	1.59	3.80	3.02	2.11	1.68	4.00	3.18
	CV(%)	49.19		35.65		10.58		26.15		32.49		21.58		17.54	
Mn	Mean	2.90	1.91	3.57	2.35	2.60	1.71	2.32	1.53	2.84	1.87	3.09	2.03	3.09	2.03
	CV(%)	29.91		33.08		29.02		26.78		56.32		14.44		20.61	
Mo	Mean	1.13	3.65	1.21	3.92	0.83	2.69	0.83	2.70	1.14	3.69	1.02	3.32	1.42	4.61
	CV(%)	40.63		31.38		16.70		24.51		38.82		28.97		40.51	
Na	Mean	0.74	0.74	1.33	1.32	0.72	0.72	0.81	0.81	0.84	0.84	0.69	0.69	0.56	0.55
	CV(%)	38.94		34.83		31.54		15.37		44.90		14.64		19.57	
Ni	Mean	0.13	1.55	0.17	2.03	0.10	1.25	0.11	1.32	0.13	1.59	0.12	1.43	0.14	1.70
	CV(%)	36.85		30.57		20.30		34.04		47.44		17.68		40.41	
P	Mean	44.54	33.63	40.40	30.51	35.32	26.67	36.41	27.50	29.97	22.63	62.17	46.95	50.73	38.31
	CV(%)	48.26		39.06		38.77		91.00		56.61		29.84		31.25	
Pb	Mean	4.65	7.88	3.34	5.67	2.93	4.97	2.82	4.79	5.36	9.10	3.38	5.74	6.94	11.77
	CV(%)	48.15		34.42		20.54		30.12		40.53		28.84		25.34	
Sb	Mean	3.10	8.87	3.85	11.01	2.70	7.73	2.50	7.16	3.08	8.81	3.70	10.58	3.08	8.82
	CV(%)	29.86		30.26		16.97		20.82		35.79		36.09		22.67	
Sc	Mean	1.74	0.74	2.06	0.88	1.65	0.70	1.66	0.71	1.73	0.74	1.89	0.80	1.66	0.71
	CV(%)	17.87		29.57		10.01		20.13		17.84		9.92		17.31	
Sm	Mean	2.32	2.04	0.92	0.81	1.67	1.47	2.16	1.90	2.88	2.54	1.95	1.72	2.96	2.61
	CV(%)	34.51		26.12		15.07		23.10		20.87		19.62		19.46	
Tb	Mean	2.24	1.86	1.09	0.91	1.56	1.29	2.11	1.75	2.64	2.20	1.88	1.56	2.90	2.41
	CV(%)	35.52		17.87		20.52		24.39		19.48		23.01		25.10	
Th	Mean	1.95	1.37	1.25	0.88	1.87	1.31	2.29	1.61	2.18	1.53	2.11	1.48	1.80	1.26
	CV(%)	31.85		38.35		16.11		21.30		40.37		19.56		37.34	
Ti	Mean	2.81	1.74	4.80	2.97	2.96	1.83	3.12	1.93	3.44	2.13	2.59	1.60	1.93	1.19
	CV(%)	43.74		32.46		18.04		39.46		56.27		16.16		13.16	
Tl	Mean	1.32	2.11	1.50	2.40	1.11	1.78	1.11	1.78	1.24	1.99	1.39	2.22	1.45	2.32
	CV(%)	29.93		50.38		17.27		28.03		38.67		20.68		26.54	
V	Mean	3.07	1.53	4.86	2.41	2.98	1.48	3.03	1.50	3.54	1.76	2.99	1.49	2.53	1.26
	CV(%)	34.24		20.80		21.90		27.06		51.27		19.27		22.63	
Zn	Mean	15.20	11.30	9.55	7.10	14.85	11.04	6.85	5.09	11.97	8.90	17.60	13.08	21.07	15.67
	CV(%)	46.36		15.13		29.47		34.97		34.85		20.84		32.82	

EF refers to enrichment factor and was calculated with Al as the reference element. UCC refers to the upper continental crust. SS refers to the local surface soil. CV refers to the coefficient of variance (defined as the standard deviation divided by the mean). EF_SS_ and EF_UCC_ had the same CV for each element in a lichen. Aluminium was not included because it is a reference element. XM refers to *Xanthoria mandschurica* (n = 5). PT refers to *Physcia tribacia* (n = 5). XE refers to *Xanthoria elegans* (n = 6). XPT refers to *Xanthoparmelia tinctina* (n = 5). PA refers to *Physcia aipolia* (n = 6). XPC refers to *Xanthoparmelia camtschadalis* (n = 10). n = 37 for all lichens.
